# Quercetin and Cisplatin combined treatment altered cell cycle and mitogen activated protein kinase expressions in malignant mesotelioma cells

**DOI:** 10.1186/s12906-016-1267-x

**Published:** 2016-08-11

**Authors:** Asuman Demiroglu-Zergeroglu, Emel Ergene, Nurettin Ayvali, Victor Kuete, Hulya Sivas

**Affiliations:** 1 Department of Molecular Biology & Genetics, Faculty of Science, Gebze Technical University, 41400 Gebze, Kocaeli Turkey; 2 Department of Biology, Faculty of Science, Anadolu University, 260470 Eskişehir, Turkey; 3 Department of Biochemistry, Faculty of Science, University of Dschang, P.O. Box 67, Dschang, Cameroon

**Keywords:** Cell cycle, Cisplatin, JNK, MAPK, Mesothelioma, Quercetin, p38

## Abstract

**Background:**

Malignant mesothelioma is a locally aggressive and highly lethal neoplasm of pleural, peritoneal and pericardial mesothelial cells without successful therapy. Previously, we reported that Quercetin in combination with Cisplatin inhibits cell proliferation and activates caspase-9 and -3 enzymes in different malignant mesothelioma cell lines. Moreover, Quercetin + Cisplatin lead to accumulation of both SPC111 and SPC212 cell lines in S phase.

**Methods:**

In present work, 84 genes involved in cell growth and proliferation have analysed by using RT^2^-PCR array system and protein profile of mitogen activated protein kinase (MAPK) family proteins investigated by western blots.

**Results:**

Our results showed that Quercetin and Quercetin + Cisplatin modulated gene expression of cyclins, cyclin dependent kinases and cyclin dependent kinases inhibitors. In addition genes involved in JNK, p38 and MAPK/ERK pathways were up regulated. Moreover, while p38 and JNK phosphorylations were increased, ERK phosphorylations were decreased after using Quercetin + Cisplatin.

**Conclusion:**

This research has clarified our previous results and detailed mechanism of anti-carcinogenic potential of Quercetin alone and incombination with Cisplatin on malignant mesothelioma cells.

## Background

Malignant mesothelioma (MM) is a neoplastic proliferation that develops from pleural, peritoneal or rarely pericardial mesothelial cells [1]. Long time exposure to asbestos and/or erionite and genetic predisposition are known to cause MM [2]. Platinum drug ‘Cisplatin’ (CIS) is conventionally employed against to MM [1]. Indeed, the combination therapies with CIS & Pemetrexed or Raltitrexed are currently used to treat MM patients [3]. However, it is required to improve alternative strategies or drugs since resistance of cancer cells to CIS therapy [4]. Quercetin (QU) is a plant derived flavonoid exhibiting anti-proliferative, growth suppressing and apoptotic effects in several cell lines [5–7]. QU induced anti-proliferative effect associated with alterations in the signal transduction pathways including MAPKs, PI3K/AKT and EGFR [7–10]. The enhanced anti-proliferative effect of QU with CIS was reported in cervix, leukaemia and hepatocellular carcinoma cells [11–13]. Additionally, we formerly reported that QU + CIS treatment of different MM cell lines (SPC111 and SPC212) caused a decrease in proliferation and an increase in apoptosis [14]. However, SPC212 cells were found to be much more sensitive to QU + CIS. Therefore, in the present work, 84 genes involved in cell proliferation and growth are evaluated in QU, CIS and/or QU + CIS treated SPC212 cells.

## Methods

### Compounds and reagents

QU and CIS (Sigma-Aldrich) stock solutions were prepared in dimethylsulfoxide (DMSO, cell culture tested; Sigma-Aldrich) and stored at −20 °C. The chemicals were diluted 100X in fresh media before each experiment. The anti-proliferative activity of single and combined chemical treatments were assessed in a monolayer culture condition by plating cells in 100 mm petri dishes.

### Cell line and culture

A human MM cell line SPC212 was used as a model system. SPC212 was derived from a tumour with mixed histology of female patient, which was obtained as a gift from The Institute of Histology and General Embryology, University of Fribourg, Switzerland. Cells were cultured in RPMI, supplemented with 10 % FBS, L-glutamine (2 mM), 2 % NaHCO_3_ (Sigma-Aldrich), and 1 % penicillin/streptomycin (Invitrogen-Gibco) in a humidified atmosphere containing 5 % CO2 at 37 °C. Cells were treated with single and/or combined concentrations of QU (50 μM) and CIS (5 μg/mL) according to our previous findings from MTS assay [14].

### RNA isolation and RT^2^-PCR

Total RNA was extracted from 2 × 10^6^ cultured SPC212 cells exposed to 50 μM QU, 5 μg/mL CIS and in combination or only % 0.01 DMSO as a solvent control for 48 h using RNeasy Mini Kit and treated with RNase-free DNase (SABiosciences, Qiagen) according to the manufacture’s protocol. Before the total RNA extractions, cell lysate was homogenized by using QIA shredder homogenizer (SABiosciences, Qiagen) and then reverse transcription of 2 μg of total RNA into cDNA was performed using the RT^2^ First Strand Kit (SABiosciences, Qiagen). cDNAs were kept on ice and then immediately used for RT-PCR array or were stored at −20 °C until using the further processes. The RT^2^-PCR assays were performed on Stratagene Mx3005P using the MAP Kinase Signalling Pathway RT^2^ profiler PCR Array (#PAHS-061Z; SABiosciences) and RT^2^ SYBR Green Master Mix (SABiosciences, Qiagen) according to the manufacture’s protocol. Thermal profile was set as segment 1 for denaturation (1 cycle): at 95 °C for 10 min; segment 2 for annealing/extension (40 cycle): at 95 °C for 15 s, at 60 °C for 1 min; segment 3 for melting curve (1 cycle): at 95 °C for 1 min, at 55 °C for 30 s, at 95 °C for 30 s. The mRNA expression levels of 84 MAP Kinase Signalling Pathway-related genes in the QU and in combination treated cells were compared to control cells, and then evaluated according to the ΔΔCt method using Data Analysis web-based software (www.SABiosciences.com/pcrarraydataanalysis.php). Data were normalized to housekeeping genes included in a RT^2^PCR Array plate (*ACTB*; β-actin and *RPLP0*; ribosomal protein large P0). Several experiments were performed and only the one with *p*-values above 0.90 for all genes after analysis using Data Analysis web-base software was considered.

### SDS PAGE/Western blotting

Cells were cultured as 2 × 10^6^ cells per 100-mm-dish incubated for 24 h, and then serum starvation is performed for the next 24 h. Later, medium was replaced with serum-free medium and cells were incubated for 12, 24 and 48 h with or without chemicals, as 5 μg/mL CIS and 50 μM QU and in combination. Then, whole-cells were obtained from PBS suspension and passed through syringes for 15 times to explode cells. Lysates were collected within 200 μL of lysis buffer and 1 % protease phosphatase inhibitor cocktail (Thermo Scientific). Protein concentrations of samples were determined by Pierce BCA Protein Assay Kit. 15 μg of total protein from each sample were separated on a 12 % SDS-PAGE and transferred to PVDF membranes (Hybond-P Amersham, GE Healthcare). Membranes were blocked 1 h in TBS-T containing 5 % non-fat dried milk that followed by application of antibodies for p-ERK and ERK (Santa Cruz Bio), p-p38, p38, p-JNK, JNK and Actin (Cell signalling) and then respective secondary antibodies conjugated with horseradish peroxidase. Thermo, Pierce ECL kit was used to develop membranes to detect chemiluminescence which is detected via ChemiDoc XRS (Bio-Rad) imaging system.

## Results

### The effects of QU + CIS on cell cycle and MAPK pathway genes

To investigate whether QU + CIS changed gene expressions of SPC212 cells, we exposed cells with QU + CIS (50 μM + 5 μg/mL) and QU (50 μM). The RT^2^-PCR array revealed that compared to untreated cells expression of cylin dependent kinase inhibitor (CDI) genes [*CDKN1A (p21), CDKN1B (p27), CDKN1C (p57), CDKN2A (p16)* and *CDKN2B (p15),*] were up regulated in QU + CIS and QU treated cells respectively.

**Fig. 1:**
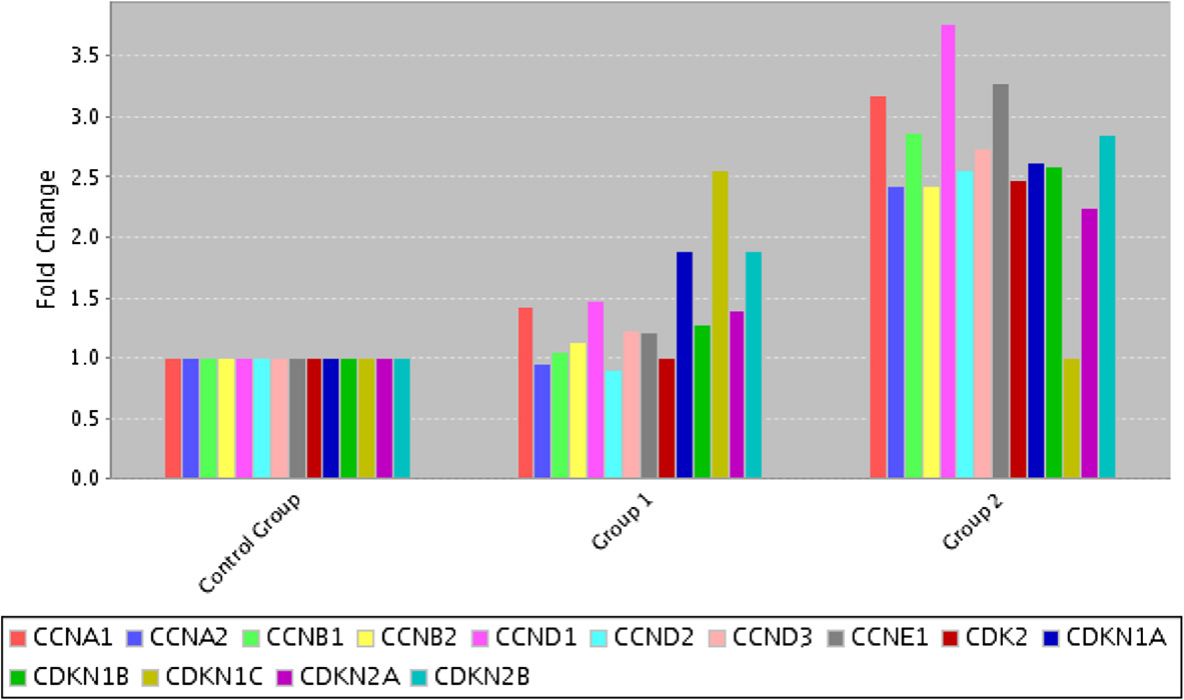
mRNA levels of cell cycle regulators. CDI, cyclin and cyclin dependent kinase(CDK) expressions in SPC212 cells treated with QU and QU + CIS. Two-fold or more differences compared to control cells were evaluated by RT-PCR array. Group 1: DMSO Control versus Quercetin; Group 2: DMSO control versus Quercetin + Cisplatin

Interestingly, cyclins [CCNA *(cyclin A1)*, CCNA2 *(*cyclin *A2),* CCNB1 *(c*yclin *B1)*, CCNB2 *(*cyclin *B2)*, *CCND1 (cyclin D1)*, *CCND2 (cyclin D1)*, *CCND3 (cyclin D3) and CCNE1 (*cyclin *E1)*] and cylin dependent kinase2 (*CDK2*) gene expressions were also elevated greater than two-fold in QU + CIS treated cells (Fig. 1).

**Fig. 2:**
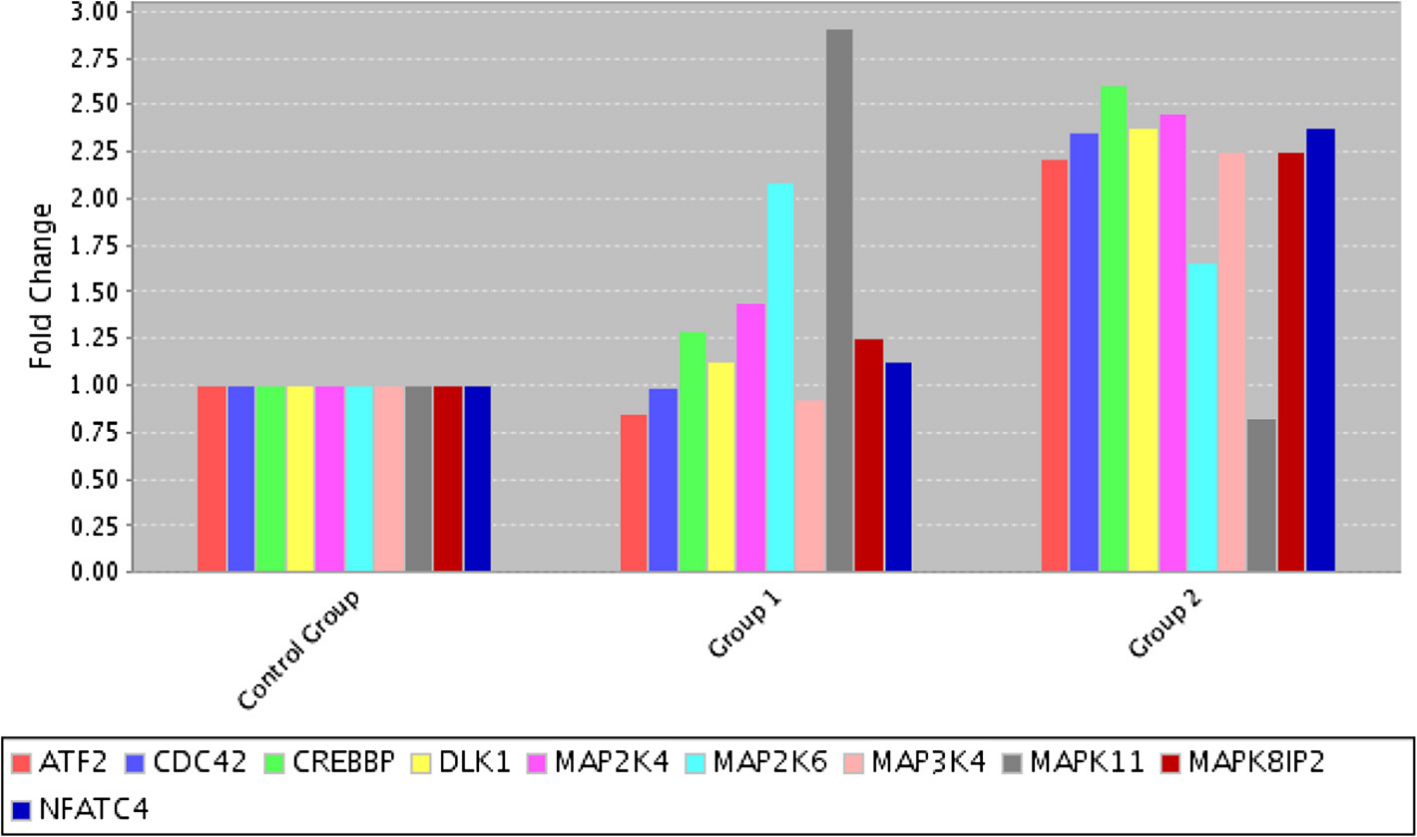
mRNA levels of JNK/SAPK and p38MAPK pathway members. Cells treated with 50 μM QU and 50 μM QU + 5 μg/ml CIS. Two-fold or more differences compared to control cells were evaluated by RT^2^-PCR array Group 1: DMSO Control versus Quercetin; Group 2: DMSO control versus Quercetin + Cisplatin

We next examined the alteration of JNK/SAPKs and p38MAPK pathway genes after drug applications. As seen in Fig. 2, treatment with QU + CIS resulted in increased expression of *DLK*, *MAP3K4 (MEKK4)*, *MAP2K4 (MEK4)*, *CDC42* and *MAPK8IP2/JIP1* (interacts with *JNK1*) genes on two-fold or more. Moreover, transcription factors (TFs) activated through JNK pathway including *ATF2 (Creb-2)*, *NFATC4 (NFAT3)* and *CREBBP* (transcriptional co-activator) were also up regulated comparing to untreated cells. Although any up regulation in the expression of genes involved in JNK pathway, arise gene expressions of p38 pathway (*MAP2K6/MEK6* and *MAPK11/p38β*) were observed in QU treated cells.

**Fig. 3:**
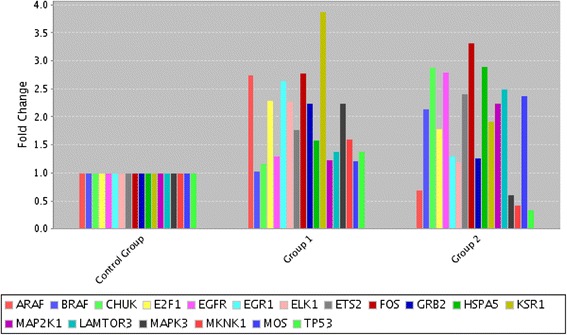
mRNA levels of MAPK and survival pathway members. Two-fold or more differences compared to control cells were evaluated by RT^2^-PCR array. Group 1: DMSO Control versus Quercetin; Group 2: DMSOcontrol versus Quercetin + Cisplatin

Similarly, genes involved in classical MAPK pathway were explored after treatment either with QU or QU + CIS (Fig. 3). Genes including *ARAF, GRB-2, MAPK3/ERK1* and *KSR-1, FOS, ELK-1, E2F1* and *EGR1* were up regulated two-fold or more in single QU treatments. Unlikely, expression of *EGFR, BRAF, MOS, MAP2K1/MEK1, LAMTOR3/MP1, FOS* and *ETS2* genes were raised in combined treatments. In addition, genes activated in response to cell protection and/or survival including *HSPA5/HSP70* and *CHUCK/IKKα* were up regulated in combine treatments. Interestingly, only two genes were down regulated after exposure to QU + CIS including MAPK interacting kinase (*MNK1*) and tumour suppressor protein, *TP53* (*p53)*.

### The effects of QU + CIS on MAPK pathway proteins

**Fig. 4:**
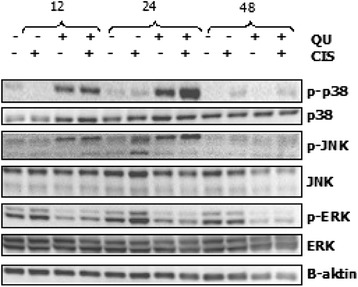
Phosphorylation and total protein levels of MAPKs. Cells were treated with 5 μg/ml CIS, 50 μM QU or both for a series of time and 15 μg their proteins were analysed by Western Blotting as described in material methods. The experiments were repeated at least three times with similar results

Finally, expression and activation levels of ERK, JNK and p38 proteins were evaluated after treatments in 48 h. As shown in Fig. 4, phosphorylation level of ERKs (p44 and p42) was decreased, but JNKs (p54 and p46) and p38 were increased in first 24 h and then, decreased (Fig. 4).

## Discussion

Development of effective inhibitors for proliferating cancerous cells is one of the most important issues in cancer treatment. Since development of drug resistance, cancer cells have been subjected to exposure chemicals especially in combinations [3]. It is well-known that the progression of cell cycle is regulated by Cylins, CDKs and CDIs in mammalian cells. Two distinct families of CDIs are classifying as p21/p27 and p16/p18. While the p21 family members are potent inhibitors to almost all CDK enzymes, the p16 family members are specific inhibitors to CDK4 and CDK6 only [15]. Earlier, we revealed that QU + CIS application caused accumulation of high percent of cells in S phase at 48 h [14]. In here, up regulated gene expression of CDIs (*CDKN1A*, *CDKN1B, CDKN2A* and *CDKN2B* after QU + CIS exposure supports our previous result and identifies which genes act on cell cycle arrest mechanism of SPC212 cells. Unexpectedly, raised expression *CDK2*, G1/S, S and G2/M cyclin genes propose resistance of cells to CIS. *CDK2* is known to play transition from G1 to S phase through S phase to G2 phase [16]. Since *CDK2* is associated with CIS resistance [17], it is an important candidate target for cancer therapy. Therefore, it may recommend integrating specific *CDK2* inhibitor into QU + CIS mix for future work. Single QU treatments in SPC212 cells caused up regulation of *CDKN1C, MEK6* and *MAPK11* gene expressions. Recently, it is reported that environmental stress transiently activates p38MAPK, which in turn, phosphorylates p57 leading an increased affinity to CDK2-CyclinE/A activity and subsequent G1 arrest [18]. Thus, this can be also the case in QU treated SPC212 cells. Besides, raised *MAPK* gene expressions may indicate maintenance of this effect.

Tanida et al. demonstrated that CIS induced toxic effects via MAPKs [19]. Zhang et al. and others [20–22] reported that ELK-1, Rac and CDC42 activated p38MAPK. In addition, Fan et al. and others [23–26] showed that DLK and MEK also activated both p38 and JNK pathways. Similarly, along with our results we assume that *CDC42*, *DLK*, *MEKK4*, *MEK4* and *JNK interacting protein* (*JIP1*) up regulations implies involvement of p38 and JNK pathways in QU + CIS exposed SPC212 cells. Besides, up regulations of TF genes including *ATF-2, NFAT* and *CREBBP* (co-activator) encouraged contribution of p38 and JNK pathways. Moreover, as it is demonstrated in Fig. 4, increased phosphorylation of JNK and p38 proteins indicated activation of both pathways in response to combined treatments. Thornton et al. [27] reported that p38 negatively regulates cell cycle by several mechanisms through down regulation of cylins, up regulation of CDI and modulation of p53. Our results propose that QU and QU + CIS mediate S phase arrest through up regulation of CDI, which may be associated with gene and protein up regulations of p38 in SPC212 cells. Beyond cell cycle arrest, activation of p38 and JNK pathways are also often correlated with stress-related apoptosis [28]. Initially we found that exposure to cells with QU + CIS caused enzymatic Caspase- 3 and caspase- 9 activations [14]. Therefore, another possibility that QU + CIS generated apoptosis of SPC212 cells as a result of activation of JNK and p38 pathways.

It is well documented that activated p53 triggers a number of signalling pathways leading to cell cycle arrest, DNA repair, apoptosis and senescence [29]. Surprisingly, *p53* gene expression was down regulated in QU + CIS treated cells. Tafolla et al. [30] exposed that activation of JNK pathway lead to negative regulation of *p53*. Thus, we thought that negative regulation of *p53* after QU + CIS treatment may related to JNK pathway activations in SPC212 cells. However, it is also possible that p38 and JNK signalling mediated apoptosis was not depended on p53 activity [31]. Indeed, analysed initial apoptotic gene expressions patterns signified that *TP73* (*p73)* gene was up regulated in the combine treatments (data not shown). In addition, QU mediated mitochondrial apoptosis was reported in the absence of p53 but in the presence of TP63 (p63) and p73 [32] and CIS activated p73-dependent apoptosis was informed by Yoshida et al. [33]. Subsequently, our results indicate existence of a p53-independent apoptosis in response to QU + CIS treatments.

Mainly, activation of MAPK/ERK pathway is associated with proliferation and survival of cells. On the contrary, increasing evidence shows that MAPK activation contributes to apoptosis in some cell types. Conflicting results exists on the consequences of CIS initiated MAPK activations. Persons et al. [34] demonstrated that CIS induced MAPK1/2 activation protects cells from cytotoxicity of CIS. However, Wang et al. [35] indicated that CIS creates MAPK initiated apoptosis. Similar contradictory results also reported on QU’s activities. Some researchers showed that apoptotic contribution of QU requires inactivation of the MAPK pathway and others declare opposite effects [7]. Our gene analysis demonstrated that although some MAPK pathway genes were up regulated, the protein phoshorylations were down regulated in QU and QU + CIS treated cells. In combine treatments another up regulated expressions was on *HSP70/HSPA5* and *IKKα/CHUCK* genes. *HSP70* occasionally was expressed in response to unfolded protein stress [36] and *IKKα/CHUCK* was induced by DNA damages or activation of NF-kappa-β pathway [37]. These results propose that QU + CIS induced stress dependent survival signals leading to increased *MAPK* gene activation. At the same time, the cytostatic and/or apoptotic capacities of agents decreased post-transcriptional phosphorylation of SPC212 cells. In addition, we observed down regulation of *MNK1* gene after QU + CIS treatments. Activation of MNKs (*MNK1* and *MNK2*) was indicated in drug resistance and negative regulation of apoptosis via modulating transcription of anti-apoptotic genes [38, 39]. Down regulation of *MNK1* gene may suggest subsequent down regulation of anti-apoptotic genes leading to apoptosis of SPC212 cells.

## Conclusions

In conclusion, QU + CIS induced S phase arrest that is regulated by almost all CDIs in SPC212 cells. The suppressed cell proliferation arrested cell cycle and triggered cell death of SPC212 cells depends on three MAPK pathway activations. Although survival /resistance pathways activated by QU + CIS treatments, SPC212 cells were undergone to apoptosis which probably implles JNK and p38 MAPK pathway involvment

## Abbreviations

cDNA, complementary DNA; CIS, cisplatin; DMSO, dimethylsulfoxide; DNA, deoxyribonucleic acid; FBS, fetal bovine serum; MAPK, mitogen activated protein kinase; MM, malignant mesothelioma; QU, quercetin; RNA, ribonucleic acid; RPMI, Roswell Park Memorial Institute 1640 medium; RT^2^-PCR, reverse transcriptase-real time polymerase chain reaction
